# Validity of the Family-Based Association Test for Copy Number Variant Data in the Case of Non-Linear Intensity-Genotype Relationship

**DOI:** 10.1002/gepi.21674

**Published:** 2012-09-12

**Authors:** Manuela Zanda, Suna Onengut, Neil Walker, John A Todd, David G Clayton, Stephen S Rich, Matthew E Hurles, Vincent Plagnol

**Affiliations:** 1 Wellcome Trust Sanger InstituteHinxton, United Kingdom; 2 Center for Public Health Genomics, University of VirginiaCharlottesville, Virginia; 3 WT/JDRF Diabetes and Inflammation Laboratory, University of CambridgeUnited Kingdom; 4 University College London Genetics InstituteLondon, United Kingdom

**To the editor**:

Ionita-Laza and colleagues have proposed a family-based association test (FBAT) based on raw intensity data from copy number variant (CNV) assays rather than genotype calls [Ionita-Laza et al., 2008. This work is motivated by the difficulty of obtaining reliable discrete CNV calls owing to the limited resolution of CNV assays, especially for complex and multi-allelic CNVs. Briefly, assuming a binary outcome phenotype and a trio design, the following score statistic was proposed for the trio *i*:

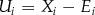


where *X_i_* is the CNV raw intensity for the affected offspring and

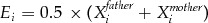


is the expectation of *X_i_*, estimated using the average intensity from both parents (i.e., a midparent intensity). The score statistic is the sum over all trios: 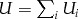. To estimate the score variance, the authors propose to use an empirical estimate 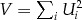, which is a Huber-White variance estimator [Huber, 1967.

An issue arises from the statement in equation 2, which is required for the expectation of the score statistic *U_i_* to be equal to 0. This result implicitly assumes that the CNV intensity is linear with the discrete CNV genotype call. If this is not the case then the expected CNV data of the affected offspring (*X_i_*) will differ from the midparent intensity, under the null hypothesis of no association and for some pairs of parental genotypes. In this non-linear case, the contribution of each trio to the score statistic *U* does not have zero expectation conditional on the genotype of the parents. Hence the test can be biased, which is likely to lead to spurious associations.

**Fig. 1:**
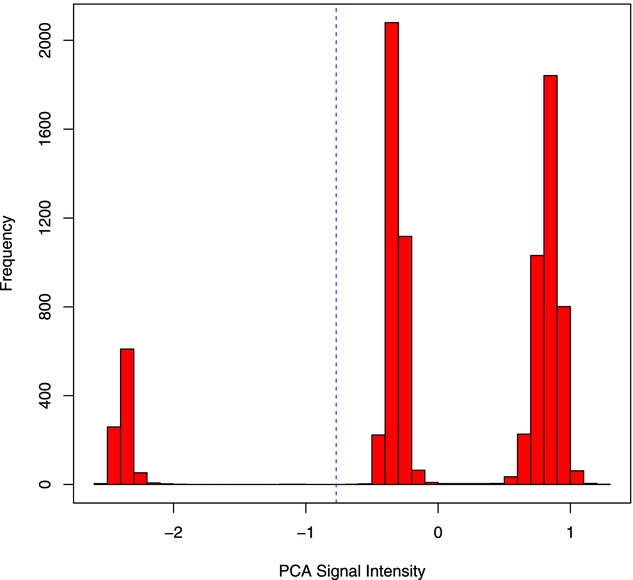
Raw CNV intensity distribution data using an array comparative genomic hybridisation (aCGH) array for *n* = 8,172 individuals at a common CNV deletion. The vertical dashed line shows the homozygous groups midpoint intensity. It is typical in aCGH data that the distance between the homozygous deletion (left most cluster, copy number 0) and the heterozygous class (middle cluster, copy number 1) is larger than the distance between the heterozygous and the normal copy number cluster (right most cluster, copy number 2). Consequently, if both parents have copy numbers 0 and 2, the offspring must have genotype 1, and the intensity in this group is systematically higher than the average of both parents.

In array-generated CNV data, it is typical to observe a non-linear relationship between CNV genotype and raw intensity data. In the example of a common deletion presented in Figure 1, for which the intensity-CNV state relationship is clearly not linear, if both parents are homozygous with copy number states 0 and 2 then the affected offspring will be heterozygous and the CNV intensity will be systematically higher than the midparent intensity (Table 1). Hence, the score statistic *U_i_* will be biased toward positive values. Conversely, if parents are both heterozygous with copy number states 1, *U_i_* will be biased toward negative values (Table 1). In fact, if the mean positions for the three clusters (copy numbers 0, 1, and 2) in Figure 1 are denoted by *a*, *b*, and *c*, and the respective frequencies of the genotypes are *f*_0_, *f*_1_ and *f*_2_, a straightforward analysis of all possible parental and offspring genotypes (Table 1) shows that the marginal expectation of *U_i_* is:

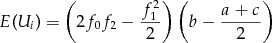

which is in general different from 0.

**Table I:** Conditional (given the parental genotypes) and marginal expectation/sign of the score statistic *U_i_* for a di-allelic deletion CNV (as shown in Figure 1). Genotype frequencies for the three copy numbers (0, 1 and 2) are *f_0_*, *f_1_*, and *f_2_*_._ The mean positions of the three clusters are *a*, *b*, and *c*. The sign computation assumes that the raw CNV intensity distributions for the three genotype classes are perfectly separated. If the genotype-intensity relationship is linear then *c* = (*a* + *b*)/2 and all the conditional expectations are equal to 0

Parental genotypes	Probability of parental genotypes	*E* (*U_i_*|parental genotypes)	*P* (*U_i_* > 0|parental genotypes)
(0,0)	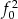	0	0.5
(0,1)	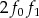	0	0.5
(0,2)	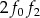	*b* − (*a* + *c*)/2	1
(1,1)	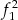	0.25 × (*a* − *b*) + 0.25 × (*c* − *b*)	0.5
(1,2)	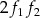	0	0.5
(2,2)	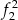	0	0.5
Marginal	1	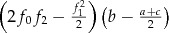	0.5 + *f_0_ f_2_*

This limitation of the FBAT CNV test was noted indirectly [Ionita-Laza et al., 2008 by mentioning that the proposed score test is robust to *linear* transformation of the intensity data. Indeed, it is the non-linearity of the copy number state/raw CNV data that is the challenge. Owing to the widespread non-linearity of the raw CNV data combined with the limitation of the FBAT CNV test, we suggest caution in interpreting these association results. Nevertheless, in their publication [Ionita-Laza et al., 2008, Figure 4], a clear example of a non-linear genotype/intensity dataset was shown for CNV Chr8tp-17E9. Remarkably, in spite of the clear non-linearity of the CNV signal, the distribution of the FBAT CNV test statistic appears consistent with its expectation under the null. This apparent robustness of the test led us to further investigate the behaviour of this test.

The analytical result for the expectation of the score statistic in the di-allelic case shown in 3 indicates that if the frequencies *f_0_*, *f_1_*, and *f_2_* are consistent with Hardy-Weinberg equilibrium (HWE), then *f_0_ f_2_* = *f_1_*^2^/4, and therefore the expectation of the score statistic is 0. An intuitive explanation for this result is the fact that, under HWE and with a large sample size under the null hypothesis of ‘no disease association’, it is known [Hardy, 1908] that subsequent generations will also be in HWE (with approximately the same allele frequency) in absence of mutation, migration and selection. Consequently, if the raw CNV intensity data depend only on the CNV genotype, then the raw CNV intensity in the offspring and parents will have the same distribution. Therefore, the score test statistic *U_i_* will have non-zero expectation conditionally on some parental intensities (Table 1), the marginal expectation of *U* (estimated in 3 by taking the weighted average of *U_i_* across all possible parental genotypes, Table 1) will be equal to 0, even if the genotype/intensity relationship is non-linear. A consequence of this general argument is that, even for more complex multi-allelic CNVs, the score statistic remains valid as long as the parental genotypes are under HWE. The result shown in 3 also shows that any population structure in the parental population that disrupts HWE also leads to a non-zero expectation for *U_i_* in the non-linear case. Hence, the robustness to population structure does not extend from the linear to non-linear case.

It is notable however that in the non‐linear case with HWE assumption, even though the marginal expectation of *U* is zero, the proportion of positive and negative values for *U_i_* is not equal in general. For example in the example of Figure 1, using the previous notations for cluster position (*a*, *b* and *c*) and genotype frequencies (*f_0_*, *f_1_*, and *f_2_*) and assuming that b > (a + c)/2 and that the genotype clusters are perfectly separated, then the same systematic check of all possible parental genotypes (Table 1) shows that

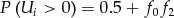
Hence, a sign test assuming equal proportion of positive and negative values for *U_i_* would not be valid.

Another issue is related to the possibility that technical biases (known as differential genotyping, [Barnes et al., 2008; Clayton et al., 2005) can create spurious differences in a case-control framework that results in false positive associations. The presence of technical bias is a primary concern of CNV association tests. This is particularly true for case-control analysis if cases and controls are recruited (and genotyped) at several sites. The trio design as developed in the FBAT CNV test can alleviate these concerns by comparing raw intensity data within families, rather than comparisons across cases and controls. In the non-linear intensity case, if the batch effect can be modelled using a family-specific covariate that acts on the raw intensity data in an additive manner, this covariate will have the same effect on the parental and offspring intensities. Therefore, the effect of this covariate will vanish in the computation of the score statistic. Thus, even in the case of a non-linear effect, the FBAT CNV statistic is expected to be robust to a range of technical artefacts.

**Fig. 2:**
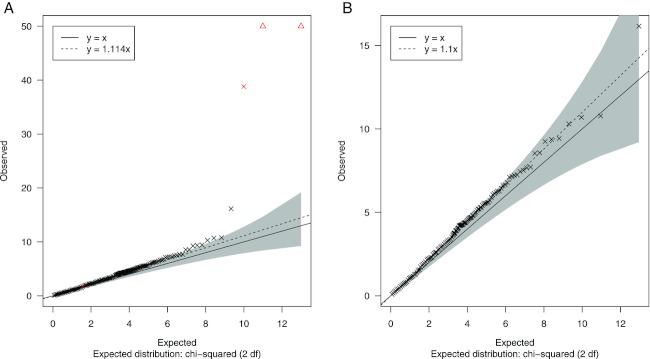
Distribution of the FBAT CNV statistic in 372 common and well-clustered CNVs computed in 2,159 multiplex T1D families (3,854 transmissions from parents to affected offsprings). The grey shaded area shows the 95% confidence interval. Panel A: All di-allelic CNVs with well-separated classes are shown. Panel B: Same as panel A but CNVs in the HLA locus (known to be T1D associated) are excluded.

To provide an example of the robustness of the FBAT CNV test in a large-scale experiment, we report in Figure 2A the distribution of the FBAT CNV test statistic for 372 common CNVs (selected on the basis of good clustering and minor allele frequency >10%) from a genome-wide CNV association scan in 2,159 T1D multiplex families (3,854 transmissions overall from parents to affected offspring). CNV intensity data were computed from a genome-wide array comparative genomic hybridisation assay (manuscript in preparation). For the association test, we used the FBAT CNV association test suggested in Ionita-Laza et al. [2008, including the robust variance estimate. Approximately 250 of these 372 CNVs are deletions for which the intensity is typically non-linear with copy number. A small subset of CNVs showed significant association (Fig.A). An inspection of these signals showed that these CNVs are all located in the HLA region, known to be T1D associated [Nejentsev et al., 2007. After removing CNVs located in the HLA region, the distribution of the test statistic was consistent with its expectation under the null (Fig.B).

In summary, the FBAT CNV score test is in general biased if the genotype-intensity link is not linear. However, although the expectation of the score statistic *U_i_* conditionally on parental genotypes may differ from zero in the non-linear case, its marginal expectation is equal to zero provided that the HWE assumption is met for the parental genotypes. In this case, the FBAT CNV test is appropriate and the initially proposed estimate of the score variance estimator *V* is consistent. The HWE assumption is not required if the genotype-intensity link is linear. Moreover, in a non-linear genotype-intensity context, the FBAT CNV test is robust to family-specific technical covariates.
